# Incidence rate of venous thrombosis in women switching combined oral contraceptives: a cohort study

**DOI:** 10.1016/j.rpth.2024.102390

**Published:** 2024-03-27

**Authors:** Deeksha Khialani, Esther de Rooij, Szimonetta Komjáthiné Szépligeti, Elena Dudukina, Saskia le Cessie, Vera Ehrenstein, Frits R. Rosendaal, Astrid van Hylckama Vlieg

**Affiliations:** 1 Department of Clinical Epidemiology, Leiden University Medical Center, Leiden, The Netherlands; 2 Department of Clinical Medicine – Department of Clinical Epidemiology, Aarhus University, Aarhus University Hospital, Aarhus, Denmark; 3 Department of Biomedical Data Sciences, Leiden University Medical Center, Leiden, The Netherlands; 4 Department of Thrombosis & Hemostasis, Leiden University Medical Center, Leiden, The Netherlands

**Keywords:** cohort studies, combined oral contraceptives, incidence rate, venous thrombosis, women

## Abstract

**Background:**

The incidence rate of venous thrombosis (VT) in women switching combined oral contraceptives (COCs) is unknown.

**Objectives:**

We hypothesize that women switching COCs may have a similar increased incidence rate of VT as women who start COCs. Switching means starting with a new COC, which may biologically approximate starting.

**Methods:**

We conducted a cohort study with data from the Netherlands and Denmark. First, we identified starters who were defined as women who did not use COCs in the 2 years prior to the start of their first COC prescription within the study period. Switchers were a subset of COC starters who redeemed a COC formulation different from their initial COC during follow-up but not longer than 12 months after starting. We estimated incidence rate ratios (adjusted incidence rate ratio [aIRR]) of VT with 95% CIs among COC switchers as compared with COC starters using Poisson regression adjusted for age, COC progestogen generation, and preexisting obesity.

**Results:**

In both countries, we found an increased risk of VT among switchers as compared with starters during the first 3 months of the follow-up (aIRR = 1.77; 95% CI, 1.22-2.56 in the Netherlands and aIRR = 1.50; 95% CI, 1.04-2.16 in Denmark).

**Conclusion:**

Switchers, particularly in the first 3 months after switching, may experience a renewed starter effect thereby increasing the risk of VT.

## Essentials

• The risk of venous thrombosis in women who switch combined oral contraceptives is unknown.
• Risk was estimated in switchers vs starters of contraceptives using Poisson regression.
• There is an increased risk among switchers vs starters during the first 3 months.
• More studies are needed to confirm this finding.

## 1 Introduction

Combined oral contraceptives (COCs) are associated with an increased risk of venous thrombosis (VT) compared with nonuse, and the risk varies depending on the type of progestogen in the COC [1]. The progestogens gestodene and drospirenone are associated with a 4-fold increased VT risk, while the progestogen levonorgestrel is associated with a 2-fold increased VT risk, all compared with nonuse of COCs [1,2].

The risk of VT associated with COC use is the highest in the first 3 months after COC initiation, ie, ∼12-fold higher compared with that associated with nonuse of COCs [3]. The risk remains 7- to 12-fold high until 12 months after initiation, after which it stabilizes, but remains 2- to 4-fold high compared with that associated with nonuse of COCs [3]. This “starters effect” is present due to the redistribution of clotting factors shortly after starting COCs, and the later risk plateau can be explained by the attrition of susceptibles [4]. Individuals at high risk of VT are likely to develop it shortly after initiating COCs, thus the number of individuals at risk of VT depletes over time.

Apart from VT, the use of certain types of COCs may have other undesired side effects, such as breakthrough bleeding, nausea, and bloating [5]. To stop experiencing these adverse effects, women may switch between different COC formulations. Furthermore, women who are using high-risk COCs, ie, those containing the progestogens gestodene or drospirenone, may switch to low-risk COCs, ie, those containing the progestogen levonorgestrel [3].

Previous literature has shown that hemostatic markers return to normal when stopping COCs [6,7]. Results of the recent study indicated that discontinuation of hormonal contraceptives results in a rapid decrease in estrogen-related thrombotic biomarkers, with 80% reduction of activated protein C (APC) resistance after 2 weeks [8]. Since biomarkers tend to normalize quickly, we hypothesized that switching COC formulation may be associated with an increased VT risk due to a new repeated redistribution of clotting factors, ie, switching may biologically approximate starting with a new COC, particularly because, when COCs are switched, there may be a practical delay between stopping and switching to another COC. This study aimed to investigate whether women switching COC formulations are at an increased risk of VT as compared with women initiating COCs.

## 2 Methods

### 2.1 Study design and study population

We conducted a cohort study using routinely collected data from 2 countries with universal healthcare access, the Netherlands and Denmark [9]. In the Netherlands, we used data from The Dutch Foundation for Pharmaceutical Statistics (SFK) [10]. In Denmark, we used data individually linked from several population-based registries: the Danish Civil Registration System [11], the Danish National Patient Registry (DNPR) [12], the Danish National Prescription Registry (NPR) [13], and the Danish Medical Birth Registry [14]. We used the DNPR to ascertain the history of comorbidities, the NPR to ascertain the COC initiation and switching, and the Danish Medical Birth Registry to ascertain the date of pregnancy start computed as the infant’s date of birth minus the gestational age at birth.

This study focused on COCs containing both estrogen and progestogen components. To increase the precision of the study results, the COCs containing the progestogens levonorgestrel, norethisterone, and norgestimate were grouped as the second-generation COCs, desogestrel and gestodene as the third-generation COCs, and drospirenone, cyproterone acetate, dienogest, and nomegestrol as the fourth/newer generation COCs (Supplementary Table S1).

In both countries, we ascertained data on women aged 10 to 49 years receiving a COC prescription between January 1, 2000, and December 31, 2016. Data on COC prescription before January 1, 2000, were unavailable.

We defined 2 cohorts: the starters and the switchers. First, we identified the cohort of COC starters, which included women who had redeemed a contraceptive prescription within the study period, ie, 2002-2016, and had no record of prescription of any form of hormonal contraceptives within 2 years before the on-study COC prescription date. Since there are no known long-lasting effects of COCs’ discontinuation [15], to align definitions of COC starters in Denmark and the Netherlands, a 2-year washout period was assumed to be sufficient for considering a woman a starter. The date of the COC initiation was the index date for women in the starters cohort. A woman was allowed to reenter the starters cohort every time she fulfilled all inclusion criteria.

The cohort of COC switchers was a subset of the COC starters who redeemed a prescription for a contraceptive formulation different from their initial COC within 12 months after initiation. The switching was allowed within the period of last prescription’s supply plus 1 month. If a woman had no COC prescription after this period, she was considered a stopper. The index date for the switcher cohort was the prescription date of the COC formulation a woman switched to.

### 2.2 Covariables

In both countries, we ascertained age at index date in both cohorts and divided it into 4 categories (10-20, 21-30, 31-40, and 41-49 years). COCs were grouped into 3 generations (Supplementary Table S1). In Denmark, there are primary and secondary diagnoses at inpatient and outpatient hospital encounters recorded in the DNPR. So we could additionally ascertain information on several comorbidities, including cardiovascular diseases, obesity, diabetes, chronic obstructive pulmonary disease or asthma, cancer, fractures or trauma, liver disease, obesity, osteoporosis, and renal failure up to 3 months (90 days) before the index date for all mentioned conditions (Supplementary Table S2). In the Netherlands, the SFK data did not provide this information.

### 2.3 Outcomes

The SFK database from the Netherlands does not provide a definitive VT diagnosis; therefore, we used treatment proxies to ascertain women’s outcomes. According to the Dutch guidelines, VT therapy before 2016 (which was the end of our observation window) consisted of a vitamin K antagonist with heparin or a direct oral anticoagulant (DOAC) preceded by heparin, depending on the type of DOAC. In accordance with these guidelines, a VT event was therefore defined as one of the following: initiation of vitamin K antagonist with any type of unfractionated heparin (UFH) or low-molecular-weight heparin (LMWH), initiation of the DOACs edoxaban or dabigatran preceded by UFH or LMWH, and initiation of the DOACs apixaban or rivaroxaban without UFH or LMWH [16]. In Denmark, we defined VT using discharge diagnoses recorded in DNPR using the date of admission. Inpatient and outpatient hospital encounters and primary and secondary diagnoses were considered (Supplementary Table S2) [17].

In both countries, women with previous VT events and VT events occurring at baseline were excluded.

### 2.4 Statistical analysis

Both starters and switchers were followed up from their index dates until VT outcome, stopping COC, death, or emigration (in Denmark), reaching 50 years as the end of reproductive age, date of pregnancy start (in Denmark), or end of follow-up at 12 months following the oral contraceptives initiation date, whichever came first. Switchers contributed to the person time calculation in the starters cohort until the time of switching. For women who switched more than one time, information after the second switch was not used. Starters contributed person time calculation in the starters cohort until the last prescription’s end of days’ supply. Similarly, switchers contributed to person time calculation in the switchers cohort until the last prescription’s end of days’ supply.

We estimated the incidence rates (IRs) per 10,000 person-years (PYs) as well as crude and adjusted IR ratio (aIRR) of VT with 95% CIs among COC switchers compared with COC starters using Poisson regression. The adjusted analyses were controlled for the confounding factors, ie, women’s age, COC generation, and a history of obesity diagnosis (in Denmark only). In both countries, we computed the IRs per 10,000 PYs and IRRs of VT in switchers vs starters by COC generation and by follow-up duration in 3-month bands (0-3, 4-6, 7-9, and 10-12 months).

In Denmark, we repeated the analyses among women aged ≤30 years since they are likely to be truly COC treatment-naive. In the Netherlands, this analysis was not carried out since women could not be followed when they changed pharmacies, and the same woman receiving COC prescriptions at 2 different pharmacies would be counted as 2 distinct women. Women aged ≤30 years often change pharmacies due to domestic migration associated with work or studies.

## 3 Results

There were 5,053,024 women starting COCs in the Netherlands over the period 2002 to 2016, among whom 153,797 (∼3.04%) switched to another COC within 12 months of follow-up. In Denmark, 855,617 women started COCs over the same period, among whom 82,929 (9.69%) switched to another COC within the 12-month follow-up. In the Netherlands, the largest proportion of women starting COCs (36.54%) were in the age category 21 to 30 years, while in Denmark, 44.97% of starters were 10 to 20 years old (Table 1). Most women switching COCs in 12 months after initiation were in the age category of 10 to 20 years in both countries (39.80% in the Netherlands and 53.85% in Denmark). The proportions of starters and switchers with obesity in Denmark were very low (0.54% and 0.18%, respectively). The proportion of women with preexisting comorbidities was also very low (≤0.50%) and similarly distributed between starters and switchers in Denmark, where data on comorbidity were available.

**Table 1:** Baseline characteristics of starters and switchers in the Netherlands and Denmark.

Netherlands		
	Starters *n* (%)	Switchers *n* (%)
**Total, *N***	5,053,024	153,797
**Age categories, y**		
10-20	1,390,214 (27.51)	61,209 (39.80)
21-30	1,846,127 (36.54)	53,781 (34.97)
31-40	1,166,696 (23.09)	27,853 (18.11)
41-49	649,987 (12.86)	10,954 (7.12)

**Denmark**		
**Total, *N***	855,617	82,929
**Age categories, y**		
10-20	384,755 (44.97)	44,660 (53.85)
21-30	208,918 (24.42)	19,374 (23.36)
31-40	195,251 (22.82)	1548 (18.67)
41-49	66,693 (7.79)	3413 (4.12)
**Obesity**^a^	4596 (0.54)	150 (0.18)
**CVD**	345 (0.04)	17 (0.02)
**Diabetes**	688 (0.08)	62 (0.07)
**COPD or asthma**	907 (0.11)	94 (0.11)
**Fractures or trauma**	5248 (0.61)	552 (0.67)
**Cancer**	231 (0.03)	15 (0.02)
**Liver disease**	104 (0.01)	≤5 (0.00)
**Osteoporosis**	47 (0.01)	≤5 (0.00)
**Renal failure**	63 (0.01)	≤5 (0.00)

COPD, chronic obstructive pulmonary disease; CVD, cardiovascular disease.

a Obesity, body mass index ≥ 30 kg/m^2^.

In the Netherlands, between 2000 and 2016, most women (74.59%) started with the second-generation COCs, while 11.00% started with the third-generation and 14.41% with the fourth-generation COCs (Table 2). In Denmark, the largest proportion of starters had a prescription for the third-generation COCs (48.30%); 33.19% of starters had a prescription for a second-generation COC, and 18.50% had the fourth-generation COC prescription (Table 3).

**Table 2:** Incidence rates per 10,000 person-years and incidence rate ratios of venous thrombosis in starters vs switchers, overall and per combined oral contraceptive generation in the Netherlands.

	Starters					
	*N* of starters (%)	*N* of VT events	Person-years	IR per 10,000 person-years (95% CI)	IRR (95% CI) switchers vs starters (ref.)	aIRR^a^ (95% CI) switchers vs starters (ref.)
Total, *N*	5,053,024	2005	3,361,434	5.96 (5.70-6.23)	Reference	Reference
Second-generation	3,769,077 (74.59)	1375	2,543,654	5.41 (5.12-5.69)	Reference	Reference
Third-generation	556,017 (11.00)	345	387,240	8.91 (7.97-9.85)	Reference	Reference
Fourth-generation	727,930 (14.41)	285	430,539	6.62 (5.85-7.39)	Reference	Reference
	***N of switchers (%)* Switchers**					
Total, *N*	153,797	70	111,241	6.29 (4.82-7.77)	1.05 (1.02-1.09)	1.27 (1.00-1.62)
Second-generation	58,036 (37.74)	18	42,781	4.21 (2.26-6.15)	0.78 (0.70-0.87)	0.91 (0.57-1.45)
Third-generation	27,110 (17.63)	17	19,178	8.86 (4.65-13.08)	0.99 (0.88-1.12)	1.72 (1.05-2.80)
Fourth-generation	68,651 (44.64)	35	49,282	7.10 (4.75-9.45)	1.07 (1.01-1.14)	1.38 (0.97-1.96)

aIRR, adjusted incidence rate ratio; IR, incidence rate; IRR, incidence rate ratio; N, number; Ref., reference; VT, venous thrombosis.

a Adjusted for age and type of combined oral contraceptives in the Total group; adjusted for age only in the different generation groups.

**Table 3:** Incidence rates per 10,000 person-years and incidence rate ratios of venous thrombosis in starters vs switchers, overall and per combined oral contraceptive generation in Denmark.

	Starters					
	*N* of starters (%)	*N* of VT events	Person-years	IR per 10,000 person-years (95% CI)	IRR (95% CI) switchers vs starters (ref.)	aIRR^a^ (95% CI) switchers vs starters (ref.)
Total, *N*	855,617	622	539,057	11.54 (10.63-12.45)	Reference	Reference
Second-generation	284,000 (33.19)	174	180,228	9.65 (8.22-11.09)	Reference	Reference
Third-generation	413,172 (48.30)	319	262,469	12.15 (10.82-13.49)	Reference	Reference
Fourth-generation	158,445 (18.50)	129	96,360	13.39 (11.08-15.70)	Reference	Reference
	***N of switchers (%)* Switchers**					
Total, *N*	82,929	61	53,576	11.39 (8.53-14.24)	0.99 (0.76-1.28)	1.11 (0.85-1.44)
Second-generation	30,215 (36.43)	19	19,517	9.74 (5.36-14.11)	1.01 (0.63-1.62)	1.07 (0.66-1.72)
Third-generation	30,832 (37.18)	24	20,013	11.99 (7.19-16.79)	0.99 (0.65-1.49)	1.12 (0.74-1.69)
Fourth-generation	21,882 (26.39)	18	14,046	12.82 (6.89-18.74)	0.96 (0.58-1.57)	1.11 (0.68-1.82)

aIRR, adjusted incidence rate ratio; IR, incidence rate; IRR, incidence rate ratio; N, number; Ref., reference; VT, venous thrombosis.

a Adjusted for age, obesity, and type of combined oral contraceptives in the Total group; adjusted for age and obesity in the different generation groups.

**Figure 1:**
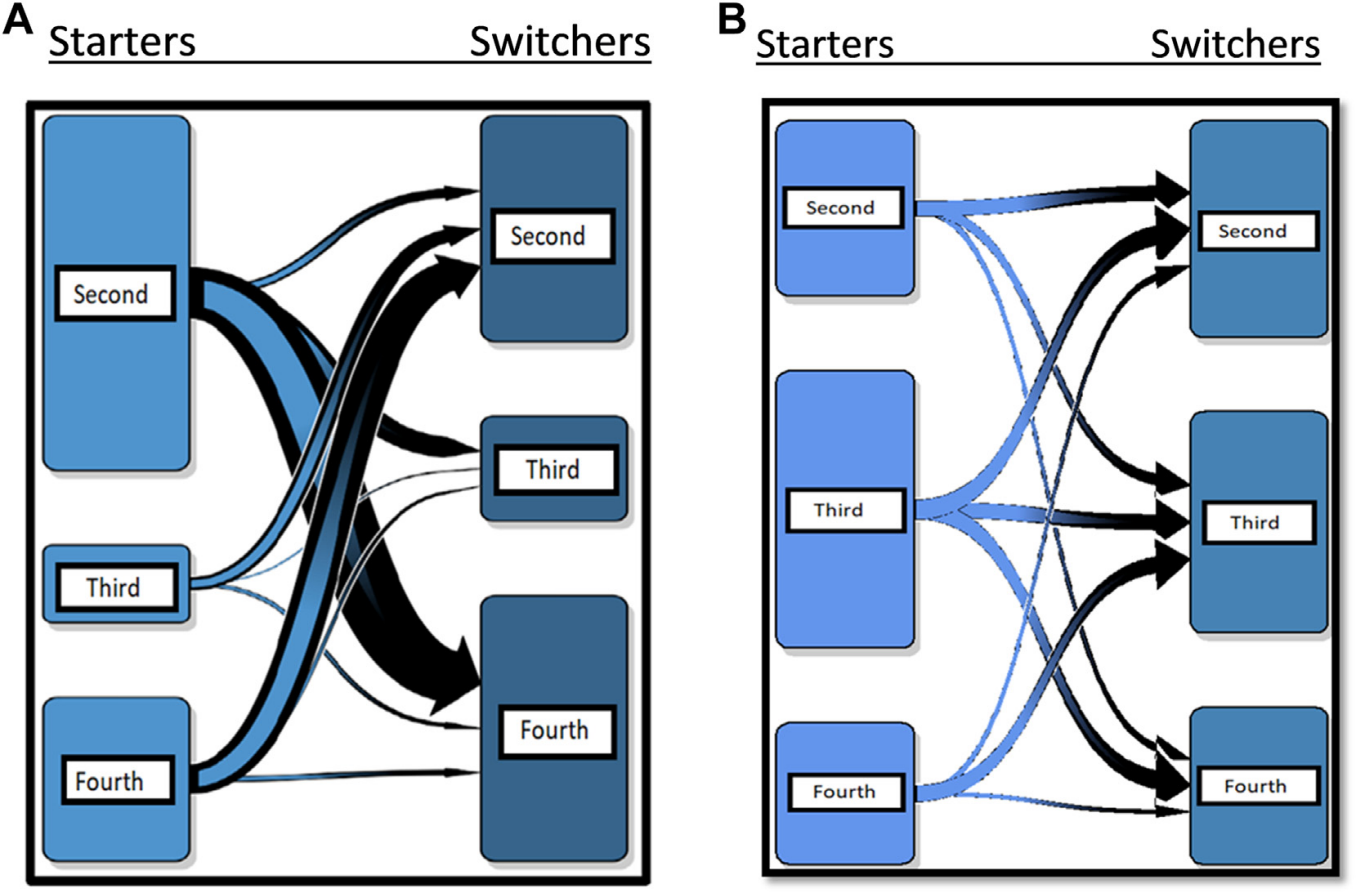
(A) Direction of switch among women starting with the different combined oral contraceptive (COC) generations in the Netherlands. Left side boxes depict the second-, third-, and fourth-generation starters. Right side boxes show the different generation switchers. Arrows indicate the direction of switch, and their thickness indicates the magnitude. Majority of the second-generation starters switched toward the fourth-generation COCs (58,196; 63.78%); the rest switched toward the third-generation (23,262; 25.50%) or the second-generation COCs (9780; 10.72%). Starters of the third-generation COCs switched mostly toward the second-generation COCs (14,430; 74.22%). The rest switched toward the fourth-generation (4087; 21.02%) or the third-generation COCs (925; 4.76%). Lastly, starters of the fourth-generation COCs switched primarily toward the second-generation COCs (33,826; 78.45%); the others switched toward the fourth-generation (6368; 14.77%) or the third-generation COCs (2923; 6.78%). (B) Direction of switch among women starting with the different COC generations in Denmark. Left side boxes depict the second-, third-, and fourth-generation starters. Right side boxes show the different generation switchers. Arrows indicate the direction of switch, and their thickness indicates the magnitude. Majority of the second-generation starters switched toward the second-generation COCs (10,727; 43.79%); the rest switched toward the third-generation (8087; 33.01%) or the fourth-generation COCs (5685; 23.21%). Starters of the third-generation COCs switched mostly toward the second-generation (14,239; 36.82%) or the fourth-generation COCs (13,433; 34.73%). The rest switched toward the third-generation COCs (11,005; 28.45%). Lastly, starters of the fourth-generation COCs switched primarily toward the third-generation COCs (11,740; 59.43%); the others switched toward the second-generation (5249; 26.57%) or the fourth-generation COCs (2764; 13.99%).

Among women starting with the second-generation COCs and subsequently switched in the Netherlands, the majority switched toward the fourth-generation COCs (63.78%). The others among the second-generation starters switched either toward the third (25.50%) or another second-generation COC (10.72%) (Figure 1A). The majority of women starting with the third- and fourth-generation COCs switched toward the second-generation COCs, 74.22% and 78.45%, respectively.

In Denmark, the majority of the second-generation starters subsequently switched toward another second-generation COC (43.79%) (Figure 1B). Starters of third-generation COCs switched mostly toward a second-generation COC (36.82%) and the fourth-generation COCs (34.73%). Lastly, starters of the fourth-generation COCs switched primarily toward a third-generation COC (59.43%).

Most of the switching occurred in the first 3 to 6 months after starting COCs in both countries (Tables 4 and 5).

**Table 4:** Number and proportion of women switching per month in the Netherlands.

Month	*N* (%)
1	14,596 (9.49)
2	20,229 (13.15)
3	27,417 (17.83)
4	12,866 (8.37)
5	15,148 (9.85)
6	14,413 (9.37)
7	8824 (5.74)
8	9814 (6.38)
9	8612 (5.60)
10	6889 (4.48)
11	7949 (5.17)
12	7040 (4.58)

*N*, number.

**Table 5:** Number and proportion of women switching per month in Denmark.

Month	*N* (%)
1	4338 (5.23)
2	8233 (9.93)
3	20,647 (24.90)
4	11,024 (13.29)
5	5405 (6.52)
6	9165 (11.05)
7	4069 (4.91)
8	4259 (5.14)
9	4943 (5.96)
10	2820 (3.40)
11	4343 (5.24)
12	3683 (4.44)

*N*, number.

The IR of VT per 10,000 PYs in starters of any COCs was 5.96 (95% CI, 5.70-6.23) in the Netherlands and 11.54 (95% CI, 10.63-12.45) in Denmark (Tables 2 and 3). The IR of VT in switchers to any COCs was 6.29 (95% CI, 4.82-7.77) in the Netherlands and 11.39 (95% CI, 8.53-14.24) in Denmark. For switchers vs starters of any COCs, the aIRR of VT was 1.27 (95% CI, 1.00-1.62) in the Netherlands and 1.11 (95% CI, 0.85-1.44) in Denmark. A similar risk pattern was seen in analyses by COC generation, albeit the number of VT events among switchers toward specific COC generation was small in both countries, resulting in wide CIs.

In both countries, the IR of VT was higher among COC switchers compared with the COC starters in the first 3 months of the follow-up, with an aIRR of 1.77 (95% CI, 1.22-2.56) in the Netherlands and 1.50 (95% CI, 1.04-2.16) in Denmark (Tables 6 and 7). In the subsequent months of follow-up, the number of VT events among switchers was low, resulting in the IRRs lacking precision.

**Table 6:** Incidence rates per 10,000 person-years and incidence rate ratios of venous thrombosis within all switchers vs all starters of combined oral contraceptives by follow-up duration in the Netherlands.

	*N* of starters	*N* of VT events among starters	Person-years among starters	IR of VT (per 10,000 person-years) in starters (95% CI)	N of switchers	N of VT events among switchers	Person-years among switchers	IR of VT (per 10,000 person-years) in switchers (95% CI)	IRR (95% CI) among switchers vs starters (ref.)	aIRR^a^ (95% CI) among switchers vs starters (ref.)
Follow-up duration										
1-3	9,851,084	739	1,204,535	6.14 (5.69-6.58)	450,748	30	36,215	8.28 (5.32-11.25)	1.35 (1.26-1.45)	1.77 (1.22-2.56)
4-6	11,494,639	547	910,875	6.01 (5.50-6.51)	347,721	18	28,061	6.41 (3.45-9.38)	1.07 (0.95-1.20)	1.20 (0.74-1.93)
7-9	8,360,085	423	666,169	6.35 (5.74-6.95)	303,168	8	24,380	3.28 (1.01-5.56)	0.52 (0.40-0.66)	0.58 (0.29-1.17)
10-12	7,013,938	296	579,853	5.10 (4.52-5.69)	266,814	14	22,585	6.20 (2.95-9.45)	1.21 (1.05-1.41)	1.43 (0.83-2.48)

aIRR, adjusted incidence rate ratio; IR, incidence rate; IRR, incidence rate ratio; N, number; VT, venous thrombosis; Ref., reference.

a Adjusted for age and type of combined oral contraceptives.

**Table 7:** Incidence rates per 10,000 person-years and incidence rate ratios of venous thrombosis within all starters vs all switchers of combined oral contraceptives by follow-up duration in Denmark.

	*N* of starters	*N* of VT events among starters	Person-years among starters	IR of VT (per 10,000 person-years) in starters (95% CI)	*N* of switchers	*N* of VT events among switchers	Person-years among switchers	IR of VT (per 10,000 person-years) in switchers (95% CI)	IRR (95% CI) among switchers vs starters (ref.)	aIRR^a^ (95% CI) among switchers vs starters (ref.)
Follow-up duration										
1-3	855,617	273	205,174.78	13.31 (11.73-14.88)	82,929	33	19,782.98	16.68 (10.99-22.37)	1.25 (0.87-1.80)	1.50 (1.04-2.16)
4-6	569,599	181	133,920.87	13.52 (11.55-15.48)	59,453	10	13,735.51	7.28 (2.77-11.79)	0.54 (0.28-1.02)	0.59 (0.31-1.10)
7-9	469,344	102	108,615.89	9.39 (7.57-11.21)	47,752	8	10,989.85	7.28 (2.24-12.32)	0.78 (0.38-1.59)	0.84 (0.41-1.74)
10-12	393,303	66	91,349.75	7.22 (5.48-8.97)	39,527	10	9068.20	11.03 (4.19-17.86)	1.53 (0.78-2.97)	1.55 (0.79-3.03)

aIRR, adjusted incidence rate ratio; IR, incidence rate; IRR, incidence rate ratio; N, number; VT, venous thrombosis; Ref., reference.

a Adjusted for age, obesity, and type of combined oral contraceptives.

Table 8 shows the IRRs of VT in switchers compared with starters by the follow-up duration in 3-month bands restricted to women of ≤30 years in Denmark. During the first 3 months of the follow-up, switchers in this age group had a IR of 9.79 per 10 000 PYs (95% CI, 4.84-14.74). The IR decreased in the follow-up months 4 to 6 (IR = 5.47; 95% CI, 1.01-9.85) and months 7 to 9 (IR = 4.50; 95% CI, 0.09-8.90) and increased in the follow-up months 10 to 12 (IR = 10.77; 95% CI, 3.31-18.24), albeit the CIs were wide. During the first 3 follow-up months, the IR of VT for switchers compared with starters was 1.24 (95% CI, 0.72-2.14). The number of VT events in switchers in the subsequent months was small, producing IRRs with wide CIs.

**Table 8:** Incidence rates per 10,000 person-years and incidence rate ratios of venous thrombosis within all starters vs all switchers of combined oral contraceptives by follow-up duration in Denmark, restricted to age ≤30 years.

	*N* of starters	*N* of VT events among starters	Person-years among starters	IR of VT (per 10,000 person-years) in starters (95% CI)	*N* of switchers	*N* of VT events among switchers	Person-years among switchers	IR of VT (per 10,000 person-years) in switchers (95% CI)	IRR (95% CI) among switchers vs starters (ref.)	aIRR^a^ (95% CI) among switchers vs starters (ref.)
Follow-up duration										
1-3	593,673	116	143,349.02	8.09 (6.62-9.56)	64,034	15	15,322.72	9.79 (4.84 -14.74)	1.21 (0.71-2.07)	1.24 (0.72-2.14)
4-6	431,958	102	101,996.87	10.00 (8.06-11.94)	47,360	6	10,965.48	5.47 (1.09-9.85)	0.55 (0.24-1.25)	0.55 (0.24-1.24)
7-9	363,258	65	84,518.76	7.69 (5.82-9.56)	38,507	≤5	Not reportable	4.50 (0.09-8.90)	0.58 (0.21-1.60)	0.58 (0.21-1.60)
10-12	308,808	35	72,070.71	4.86 (3.25-6.47)	32,257	8	7426.37	10.77 (3.31-18.24)	2.22 (1.03-4.78)	2.17 (1.00-4.71)

aIRR, adjusted incidence rate ratio; IR, incidence rate; IRR, incidence rate ratio; N, number; VT, venous thrombosis; Ref., reference.

a Adjusted for obesity and type of combined oral contraceptives.

## 4 Discussion

In this study, we investigated the risk of VT in women switching to different COC formulations in the Netherlands and Denmark compared with the risk among starters. COC switchers had an increased risk of developing VT in the subsequent 12 months compared with starters in the Netherlands (aIRR = 1.27; 95% CI, 1.00-1.62) and in Denmark (aIRR = 1.11; 95% CI, 0.85-1.44); however, for the latter country, the magnitude of an association was smaller with wider CIs. In both countries, we found an increased risk of VT among switchers as compared with starters during the first 3 months of the follow-up (aIRR = 1.77; 95% CI, 1.22-2.56 in the Netherlands and aIRR = 1.50; 95% CI, 1.04-2.16 in Denmark). Similarly, when investigating the association among women aged ≤30 years in Denmark, switchers compared with starters were at an increased risk of VT in the first 3 months of the follow-up; however, the CI was wide (aIRR = 1.24; 95% CI, 0.72-2.14).

This was a population-based cohort study conducted in 2 countries covering a data availability period of 17 years. The data were routinely collected in both the Netherlands and Denmark.

Danish data allowed uninterrupted follow-up of the COC users from the therapy initiation until the COC use cessation and switching, the outcome of interest, death, or emigration, therefore, virtually eliminating the loss of follow-up and associated with selection bias. The coding of VT in the DNPR was previously validated and showed a positive predictive value of 86% to 90%, leaving a small proportion of false-positive VT events present in analyses of Danish data [17].

According to previous research, the risk of VT among COC starters was particularly increased in the first 3 to 12 months following the contraceptive initiation [3]. Due to various side effects (breakthrough bleeding, nausea, and bloating), which are unlikely risk factors of VT [5], women change to a different COC formulation in the first months of starting oral contraceptives. We hypothesized that switching, which essentially is starting with a different COC formulation, may biologically approximate starting a different COC formulation. This may lead to redistribution of clotting factors and increase the VT risk in women. At the time of writing, there have been no studies investigating the risk of VT among COC switchers, therefore a comparison of our results with other studies was not possible.

### 4.1 Limitations

This study has several limitations. Firstly, the databases started in 2000, meaning that lifetime prescriptions of the women were not available. The starters cohort in this study may include a proportion of women who had a prior history of COC use and are therefore not first-time-starters. This could lead to an underestimation of the VT incidence among starters in both countries. In the Netherlands, an underestimation in the VT incidence among starters may also stem from the inability to follow women when changing pharmacies, which may have caused misclassification of continuous COC users with low VT risk as starters when they change pharmacy. VT incidence in potentially nontrue starters may be underestimated and may lead to a slight overestimation in the point estimate when comparing switchers vs starters. Switchers themselves also experience attrition of susceptibles since they had to be starters of COCs first before they joined the switcher’s subset. In theory, the switchers should be at a similar or slightly decreased risk of VT (because of attrition of susceptibles) compared with starters. An aIRR of >1 implies that switchers do have an increased risk of VT, similar to what is seen in starters of COCs. However, in this study, the risk may be overestimated because true starters could not always be identified.

Secondly, the Dutch SFK database does not collect VT diagnoses. We used a medication proxy when ascertaining VT outcomes; therefore, misclassification of the outcome is possible. However, since we include all treatment options for VT [16], misclassification is unlikely.

Since the SFK only contains data from the community and not hospital pharmacies, women receiving heparin in the hospital, including cancer-associated VT or pulmonary embolism as indications, could not be identified in this study. However, this number of missed VT is likely to be small since cancer and pulmonary embolism are rare in young women.

Another limitation is that different outcome definitions were available in both countries, resulting in the inability to perform a combined analysis and, therefore, relatively small sample sizes in subgroup analyses.

To account for the limitation of potentially having included false first-time starters, we performed a sensitivity analysis in women ≤30 years of age in Denmark. Most women in this young age group are likely to be true first-time starters. Results for this analysis only marginally differed from the overall results in Denmark. This analysis could not be performed in the Netherlands.

The IR of VT among starters in Denmark was 13.31 per 10,000 PYs, whereas in the Netherlands, it was 6.14 per 10,000 PYs in the first 3 months after starting. An explanation of a higher IR in starters of COCs in Denmark compared with the Netherlands may be that the majority of starters in the Netherlands are initiating with a second-generation COC, whereas, in Denmark, the majority of starters are initiating with a third-generation COC. Another possible explanation for the difference in IR of VT in both countries may be that Denmark captures more VT events than the Netherlands (no in-hospital capture).

An increased IR of VT in the first months after starting and decreasing afterward was seen in Denmark (also after restricting to starters aged ≤30 years); however, this pattern was not as clearly seen in the Netherlands.

Previous literature has shown that hemostatic markers return to normal when stopping COCs [6,7]. Unfortunately, it was not possible to investigate the IR of VT in women who had stopped COCs for a short time and then restarted. This is because, in both databases, we lacked precise data on refilling after gaps.

We compared switchers vs starters by the duration of follow-up in 3-month bands; however, we could not compute IRRs by follow-up duration in 1-month bands due to sparse data. The number of VT events among switchers after the initial 3 months of the follow-up was small and the IRRs comparing switchers vs starters became imprecise in both countries. It would be interesting to examine the relative risks by COC generations, but currently, it is not possible due to sparse data.

For confounding control, we adjusted for women's age at COC initiation and the generation of prescribed COCs along with the obesity diagnosis in Denmark only. We had no data on the reasons leading up to COC switching in women and whether or not these can be shared causes of VT. Although we cannot exclude some residual confounding being present in this study, reasons often reported for switching COCs, eg, breakthrough bleedings, nausea, and bloating, are likely unrelated to VT risk. Furthermore, 90 days before the index date, both COC starters and switchers had an equal distribution of VT risk factors, eg, diagnosed preexisting chronic condition or having had fractures or trauma.

In conclusion, this study found that women switching a COC formulation, particularly in the first 3 months after switching, may experience a renewed starters effect thereby increasing the risk of VT. More studies are needed to confirm this finding.
