# Supplementary material for: Incidence rate of venous thrombosis in women switching combined oral contraceptives: a cohort study
Source: Res Pract Thromb Haemost. 2024 Mar 27;8(3):102390. doi: 10.1016/j.rpth.2024.102390 (PMC11060944; doi:10.1016/j.rpth.2024.102390)
Supplement: Supplementary Table S1 [file mmc1.docx]

**Table S1.The medication (ATC) codes used to ascertain the starters and switchers of combined oral contraceptives**

| **Second Generation COC** |  |
| --- | --- |
| Levonorgestrel+EE | ATC: G03AA07; G03AB03 |
| Norethisterone+EE | ATC: G03AA05; G03AB04 |
| Norgestimate+EE | ATC: G03AA11 |
|  |  |
| **Third generation COC** |  |
| Desogestrel+EE | ATC: G03AA09; G03AB05 |
| Gestodene+EE | ATC: G03AA10; G03AB06 |
|  |  |
| **Fourth/Newer generation COC** |  |
| Nomegestrol+estradiol | ATC: G03AA14 |
| Dienogest+estradiol | ATC: G03AB08 |
| Drospirenone+EE | ATC: G03AA12 |
| Cyproterone acetate+EE | ATC: G03HB01 |

COC, Combined Oral Contraceptive; EE, Ethinylestradiol
