# Supplementary material for: Incidence rate of venous thrombosis in women switching combined oral contraceptives: a cohort study
Source: Res Pract Thromb Haemost. 2024 Mar 27;8(3):102390. doi: 10.1016/j.rpth.2024.102390 (PMC11060944; doi:10.1016/j.rpth.2024.102390)
Supplement: Supplementary Table S2 [file mmc2.docx]

**Table S2. Diagnostic (ICD-10) and medication (ATC) codes used to ascertain comorbidity history and VT outcomes in women, Denmark**

| **Comorbidities** |  |
| --- | --- |
| Cardiovascular diseases | ICD-10: I05-I09, I10-I15, I20-I25, I50 |
| ACE inhibitors | ATC: C09 |
| Beta-blockers | ATC: C07 |
| Aspirin | ATC: B01AC06, N02BA01 |
| Clopidogrel | ATC: B01AC04 |
| Statins | ATC: C10AA, B04AB01 |
| Calcium channel antagonists | ATC: C08 |
| Antihypertensive drugs | ATC: C02 |
| Diuretics | ATC: C03 |
| Diabetes | ICD-10: E10, E11, H36.0; ATC codes: A10A, A10B |
| COPD or asthma | ICD-10: J41, J42, J43, J44, J45, J46; ATC: R03 |
| Cancer | ICD-10: C00-C99 |
| Fractures or trauma | ICD-10: S00-T14 |
| Liver disease | ICD-10: K70.0, K70.3, K71.7, K73, K74, K76.0, B18, I85 |
| Obesity | ICD-10: E65-E68 |
| Osteoporosis | ICD-10: M80-M82 |
| Renal failure | ICD-10: N17-N19 |
| **Outcomes** |  |
| Deep venous thrombosis | ICD-10: I80.1–I80.3 |
| Pulmonary embolism | ICD-10: I26.0, I26.9 |
| Abbreviations: ICD, International Classification of Diseases; ATC, Anatomical Therapeutic Chemical classification system; ACE, angiotensin-converting enzyme; COPD, chronic obstructive pulmonary disease. | |
